# What should patients learn? Co-designing patient education to improve medication safety, professional-patient communication, and partnership

**DOI:** 10.3389/fmed.2025.1631606

**Published:** 2025-09-10

**Authors:** Z. Noah Hendrix, Fatoumata Jallow, Kimberly G. Fulda, Richard A. Young, Anna M. Espinoza, Ayse P. Gurses, Annesha White, Yan Xiao

**Affiliations:** ^1^College of Architecture, Planning, and Public Affairs, University of Texas at Arlington, Arlington, TX, United States; ^2^College of Nursing and Health Innovation, University of Texas at Arlington, Arlington, TX, United States; ^3^North Texas Primary Care Practice-Based Research Network (NorTex), Texas College of Osteopathic Medicine, University of North Texas Health Science Center, Fort Worth, TX, United States; ^4^Family Medicine, JPS Health Network, Fort Worth, TX, United States; ^5^Armstrong Institute for Patient Safety and Quality, Johns Hopkins School of Medicine, Baltimore, MD, United States; ^6^College of Pharmacy, University of North Texas Health Science Center, Fort Worth, TX, United States

**Keywords:** medication safety, patient education, partnership, communication, co-design, patient work system, information asymmetry, shared decision-making

## Abstract

**Introduction:**

Adverse drug events are a major focus of patient safety research, but work is often limited to healthcare professionals’ actions and inpatient populations. The patient work system provides a framework to understand the work done by patients and other nonprofessionals. The authors aimed to improve medication safety through the development of short educational videos designed to facilitate professional-patient partnership and shared decision-making by addressing the knowledge gaps and information asymmetry that serve as barriers to productive primary care encounters.

**Methods:**

The authors first performed a narrative review to identify knowledge gaps and the most important medication management principles for patients to learn. Next, the authors conducted participatory design workshops with professionals and patients to develop a list of topics for the educational videos. Lastly, the authors surveyed professionals (*N* = 44) and patients (*N* = 100) to measure interest in the proposed video topics.

**Results:**

The narrative review identified two themes: (1) knowledge-based barriers and hazards, and (2) opportunities for education-based solutions. The design workshops resulted in a proposed list of 12 educational videos divided into four modules: ownership, partnership, system, and learning. Two-factor ANOVA testing of the survey results showed that there was a significant difference in interest with professionals being more interested than patients (*p* < 0.001). Post-hoc testing revealed that patients were significantly more interested in watching videos from the partnership module than from the system module (*p* < 0.05).

**Discussion:**

Information asymmetry provides a framework to understand why some patients defer decision-making to professionals while also showing the greatest interest in the partnership module. It also highlights why it is important for professionals to tell patients about their desire for patient ownership of care—engaged patients provide better information to professionals, who might otherwise work with incomplete records. To improve medication safety, patient education efforts should include a focus on how patients can partner with HCPs and be mindful of the work system barriers that patients will encounter while performing the educational work. Successful efforts stand to improve patient outcomes by reducing information asymmetry and enabling shared decision-making.

## 1 Introduction

The 2000 *To Err is Human* report (1) illuminated the complex and critical issue of patient safety, with medication safety emerging as an important focus. Adverse drug events (ADEs) account for approximately 4.5 million ambulatory care visits (3) and 1.27 million emergency department visits annually (4). ADEs are not a direct measure of safety (5), but their prevalence has led to recognition of this problem and an explosion of research on their causes. Much of that research has focused on healthcare professionals’ (HCPs) actions—prescribing, dispensing, and administering—and has often emphasized inpatient care (5–7).

The last quarter century has also seen developments like the patient work system (PWS) that extended our understanding of medication management beyond the actions of professionals. The PWS examined the work done by patients and other nonprofessionals to develop a model that described the four types of barriers patients face beyond the healthcare they receive: task, tool, person-related, and context (8). The PWS is one explanation for why studies have shown that most medication-related problems are not identifiable by looking at medical charts without patient involvement (9) and why measures like potentially inappropriate prescribing are poorly correlated with the actual occurrence of ADEs (5). Patient-facing factors, professional-patient communication, and partnership are important parts of the solution to medication errors. Indeed, evidence has shown that nonadherence and ADEs are more likely among patients that: have a poor understanding of treatment regimens (10), hold negative attitudes and beliefs (10), lack medication management systems (11), experience poor communication with their primary care professionals (12), face inconvenience factors to obtain their medications (11, 13), and possess low health literacy (14).

A common theme among those patient-facing factors impacting medication safety is the opportunity for improvement through patient education. While healthcare professionals undergo years of structured training, including for soft skills like communication (15, 16), patients are often left to learn about their medications and how to manage them through fragmented interactions and scattered resources. The high levels of information asymmetry (IA) in primary care encounters are further exacerbated by time limitations that make it difficult for professionals and patients to share the important information that each brings to the interaction. IA discourages patients from seeking and sharing important information, opting instead to wait for direction from professionals (17). Therefore, closing the non-technical knowledge gaps between professionals and patients is an important part of patient-centered care (18). Digital health information tools like videos have been found to reduce IA and improve the co-creation of healthcare (19).

We sought to improve medication safety through the development of short educational videos designed to facilitate professional-patient partnership and shared decision-making by addressing the knowledge gaps and information asymmetry that serve as barriers to productive primary care encounters. We adopt the Chronic Care Model’s (CCM) framework that “activated, informed patients” are essential to productive primary care interactions and that the primary care system is designed for “brief, didactic patient education” (20). The CCM provides a link from education to more informed patients, to productive professional-patient interactions, to improved clinical outcomes. This paper describes our participatory design process to develop video topics and test whether they interest professionals and patients. Drawing on prior studies of patient education, the videos were designed to be positive (21), tailored to their audience (14, 22), and short enough to be deployed during waiting periods (21, 23).

## 2 Materials and methods

**Figure 1:**
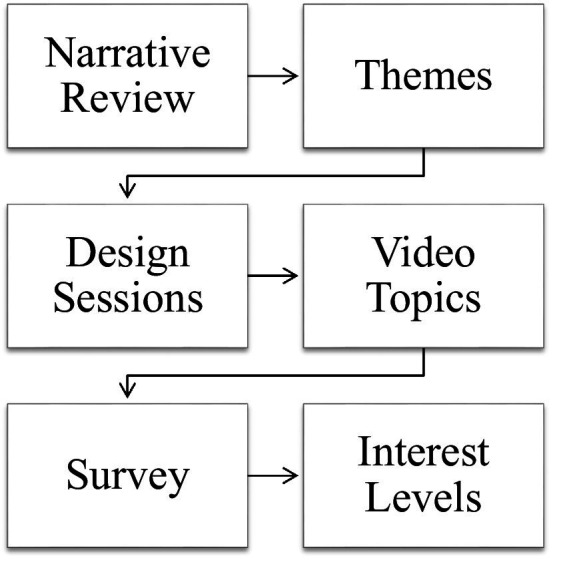
Methods overview.

This work was performed as part of the Partnership in Resilience for Medication Safety (PROMIS) Patient Safety Learning Lab (24). The authors used a three-step design process to develop the video topics: a narrative review to understand knowledge gaps and educational solutions, design sessions to develop video topics, and a survey to measure interest. Figure 1 provides an overview of the design process and shows how the results of each step served as the basis for the next one.

### 2.1 Narrative review

We began the design process with a narrative review. While previous studies have focused on the role of healthcare professionals’ actions in medication safety, this review uniquely addressed the patient perspective and the potential for patient education to bridge knowledge gaps. We searched PubMed for articles with terms like medication management, primary care, medication safety, patient education, health literacy, and knowledge gaps published from 2005 through 2022. Articles were selected based on relevance to the PWS, IA, and patient education in medication safety among older adults in the digital era, but we did not follow a specific protocol for inclusion. Several PROMIS Lab articles under development at the time were also included. Selected articles were synthesized in an iterative grouping process based on the type of knowledge gap (e.g., health literacy, misunderstood roles, incorrect EHR information, etc.) and whether the article examined a barrier or solution.

### 2.2 Design sessions with professionals and patients

The narrative review resulted in two themes: (1) knowledge-based barriers and hazards, and (2) opportunities for education-based solutions. The themes and their sub-themes served as the foundation for the design sessions with professionals and patients. The goals for the sessions were to validate the narrative review’s findings, assess informational needs (18), and organize the learning material into discrete video topics. These sessions were based on co-design methodologies that serve to authentically engage professionals and patients and avoid tokenism (25). For example, participants were presented with case scenarios of knowledge-based barriers to open discussion about their own experiences managing medications in an ambulatory primary care environment. They also engaged in “rapid prototyping” where they were shown videos topics that the research team put together and were then asked to iterate different versions of the video topics and reorganize content. We held four sessions with professionals (two at each participating site) and three with patients (one at each site plus a combined follow-up). Each design session opened with a case scenario to initiate discussion. Then participants were asked about their experiences with the sub-themes, which sub-themes were most harmful, and which were most fixable. Participants for the design sessions were recruited from two primary care clinics in the Dallas-Fort Worth metroplex (one public clinic and one private practice). The inclusion criterion for professionals was experience practicing in an ambulatory primary care clinic as a physician, physician’s assistant, nurse practitioner, nurse, or certified medical assistant. For patients, the inclusion criteria were being 50 years of age or older, taking five or more prescription medications, and speaking English or Spanish. Caregiving family members of the patients were also invited to participate.

### 2.3 Survey of professionals and patients

The design workshops resulted in a proposed list of 12 educational videos divided into four modules: ownership, partnership, system, and learning. In the third and final step of the design process, the authors took the proposed list of video topics (not the actual videos) and tested them for interest among professionals and patients. A survey was developed that asked patients whether they would watch the videos while waiting to see their primary care professional with yes/maybe/no response options for each topic. Professionals were asked if they would want their patients to watch the videos with the same response options. A free-response question was included so that participants could share if there were other important medication management lessons for patients to learn that were not covered by the proposed video topics. The surveys had six questions, excluding demographics, and took 5 to 10 min to complete. The professional survey was emailed to all primary care physicians, physician assistants, and nurse practitioners at the same clinics from the design sessions (94 total yielding 44 responses). To aid the recruitment of patients, the private practice was replaced with a second public clinic, and a convenience sample of 100 patients was recruited from the clinics’ waiting rooms. The inclusion criteria for the patient survey were 18+ years of age and English-speaking. For analysis, the videos were grouped into modules and the interest levels of the videos (no = 0, maybe = 1, yes = 2) were combined to yield an interest score for the modules (ranging 0–6). A two-factor ANOVA was used to test for differences between modules and roles (professional or patient) with a *p*-value of 0.05. Tukey’s procedure was used for post-hoc testing. Deductive thematic analysis was used to evaluate the free-response answers.

## 3 Results

### 3.1 Narrative review

Twenty-three articles were chosen for inclusion in the narrative review. Two themes emerged during the coding process. The first was knowledge-based barriers and hazards. The second was opportunities for education-based solutions. The barriers and hazards identified in Theme 1 can manifest on the patient side in three ways, which are reflected in the first three sub-themes. They are also present on the professionals’ side, as shown in the fourth sub-theme. Table 1 shows the four sub-themes for Theme 1 with example hazards.

**Table 1:** Theme 1: Knowledge-based barriers and hazards.

Theme 1: Knowledge-based barriers/hazards	Example hazard
Sub-Theme 1.1: Lacking medication management knowledge and skills	Articles: 4, 8, 9, 25, 26, 27, 28, 29, 30, 31
Not knowing basics of medication (name, dose, frequency, indication)	A patient cannot say which anticoagulant they are taking
Poor organization and management	A patient does not use an organizer and forgets whether they have taken their medication
Self-administration errors	An elderly patient finds it difficult to swallow a sustained-release tablet, so they crush it
Inappropriate changes	A patient does not feel sick anymore so they do not finish an antibiotic
Sub-Theme 1.2: Misunderstanding their role and its importance	Articles: 13, 32, 33, 34
Offloading decision-making to professionals	A patient does not tell their HCPs about side effects because they think the HCP knows best
Not understanding their role as an information provider	A patient does not tell their primary care professional about meds from specialists
Sub-Theme 1.3: Inability to navigate healthcare system	Articles: 10, 12, 13, 25, 28
Discontinuity of care	A patient receives the same prescription twice from different professionals
Using multiple pharmacies	Pharmacists are unable to check for and advise on potential drug interactions
Sub-Theme 1.4: Inaccurate or incomplete information for professionals	Articles: 4, 8, 12, 13, 28, 29, 34
Inaccurate medication lists	A professional does not know that a patient stopped taking a medication
Incomplete information about a patient’s experience	A professional does not know that a patient skips doses of medication causing fatigue

First, patients may lack the knowledge and skills needed to manage their medications effectively (sub-theme 1.1). A systematic review found that most preventable ADEs occur on the patient side (5). Complex medication regimens, comorbidities, and changes to a regimen make management an understandably difficult task for patients (10, 26–28). Some patients further increase complexity and risk by using medications prescribed to others (e.g., family members) and taking expired medications (29). Patients may also discontinue a medication or skip doses on their own without consulting professionals (30). These behaviors are often the result of limited health literacy (9). Patients also report that they do not receive all important information about their medications from their professionals (31, 32). Many patients, especially older adults, face cognitive and sensory limitations (10, 26, 29, 31). Efforts to overcome those limitations may result in self-administration errors, like when a patient that has trouble swallowing pills inappropriately splits sustained-release tablets without realizing it presents a safety risk (10, 28).

The second sub-theme (1.2) was that some patients misunderstand their role in medication safety and its importance. Patients know that they are responsible for taking their medications, but often “perceive their roles in medication safety as independent of health professionals’ roles” and “assume a passive role in shared decision-making” (33). These beliefs are barriers to partnership. One study that interviewed healthcare professionals found that their relationships with patients can be damaged when patients offload their responsibility onto professionals (34). Another study identified limited shared decision-making processes as a safety vulnerability (14). This sub-theme is strongly connected to IA. Patients realize that HCPs have more medical knowledge and elect to defer to professionals’ decision-making. Patients may also assume that their professionals have complete knowledge of their medication regimens and that information flows well throughout the healthcare system and therefore do not realize that providing information to professionals is a critical part of their role (35).

The third sub-theme (1.3) was that patients find it difficult to navigate the healthcare system. Many patients use multiple pharmacies, do not consistently see the same HCPs, and fail to follow through on prescriptions and tests. Several studies found that using multiple pharmacies presented safety vulnerabilities, increased rates of nonadherence, and led to more drug–drug interactions (11, 13, 14). One study speculated that these harms could be the result of worsened information sharing in the forms of inconsistent medication records and drug counseling from professionals, especially pharmacists (13). In a similar vein, other studies showed that discontinuity of care was also a safety vulnerability that led to regimen complexity and ADEs (14, 26, 29).

Knowledge gaps also create barriers on the professional side and are represented in sub-theme 1.4: inaccurate or incomplete information for professionals. The articles supporting this sub-theme showed that many drug-related problems and potential drug–drug interactions cannot be identified by chart review alone due to missing information (9, 29). A study of Dutch geriatric outpatients found that there was at least 1 discrepancy between medication lists held by patients, physicians, and pharmacists in 87% of cases (29). Some patients make changes to their regimes without informing their professionals (30). Professionals acknowledge the importance of communicating with patients to gain full information, but also express distrust of medication lists provided by patients (35). Other communication issues between clinics, pharmacies, and insurance companies contribute to safety vulnerabilities as well (14), especially for patients that use multiple pharmacies (which includes 38.1% of Medicare Part D beneficiaries) (13). Furthermore, professionals are sometimes overloaded with information like electronic health record messages that cause “alert fatigue” and lead HCPs to ignore potentially important safety warnings (5, 14).

The second theme was opportunities for education-based solutions. Learning points for patients could be grouped into three sub-themes: safety practices at home, communication and partnership; and how patients can learn about their medications. Table 2 details the sub-themes with examples.

**Table 2:** Theme 2: Education-based solutions.

Theme 2: Education-based solutions	Example learning point for patients
Sub-Theme 2.1: Safety practices at home	Articles: 10, 12, 26, 27, 29, 31, 34, 35, 36, 38
Adherence	Do not stop medications or skip doses without speaking to provider
Organization	Use a medication list to keep track of when you take medications and what they are for
Complexity reduction	Use one pharmacy to pick up all prescribed medications
Sub-Theme 2.2: Partnership	Articles: 13, 30, 32, 34, 37, 39, 40
Information sharing	If you have a medication list, show it to your doctor so they have up-to-date information
Visit preparation	Writing down questions before your visit helps you make the most of your appointment time
Sub-Theme 2.3: Learning about your medicines	Articles: 29, 41
Learn the basics of your medications	Learn the names of your medications, what they are for, and how to take them
Learn about your responsibilities	Doctors and pharmacists are experts, but you have responsibility outside the doctor’s office
How to learn about medications	Social media is not a reliable source of information

The first sub-theme (2.1) focused on safety practices at home. Studies included in the review reported that 40–70% of patients do not use any kind of organization system for their medications (32, 36). A systematic review found that self-monitoring and self-management programs show general effectiveness for adherence, ADEs, and clinical outcomes (37). Pillboxes and medication lists are commonly used tools for organization. Medication lists help patients take medications on schedule, check for errors, remember to order refills, and track whether medications are effective (30, 35, 38, 39). Pillboxes may be even more effective for improving adherence, but they can also cause errors if set up incorrectly (10, 11, 14, 26). The method of organization may be less important than a patient’s satisfaction with the method; one study found that organization satisfaction was associated with adherence independently of the method used (11). Our review also found that there is a need for education on complexity factors (28), like dosing (e.g., how and when to split medications) (10, 28), using a single pharmacy (13), storing medications in one place in the home (27), and disposing of expired and discontinued prescriptions (27, 36).

The second sub-theme (2.2) included education focused on how patients can productively partner with professionals. Collective ownership of medication safety through partnership is important to engage patients in their care (14). Qualitative studies show that healthcare professionals feel a fiduciary responsibility for their patients’ safety (35, 40) and that patient education can be used to expand patients’ understanding of their own role in safety (33). Education initiatives that encourage patient engagement have shown promise for reducing inappropriate medication use (38). For example, many patients that use medication lists do not show them to their HCPs unless asked (35). Proactive sharing of the list can improve information flow (35) and help professionals monitor hazards (31). Patients can also be educated on how to prepare for visits with professionals. A quality improvement project that implemented a 15-min preparation program reported that professionals and patients both believed it added value to the visits (41).

Our final sub-theme (2.3) focused on how patients can learn about their medications on their own. Patients do not see their HCPs often, so there is a need for them to self-educate between visits (30). Patients will experience situations where they are unsure how to take their medications, which presents safety risks, especially for conditions like diabetes (42). It is important that they are aware of helpful resources and able to identify trustworthy information online.

### 3.2 Design sessions with professionals and patients

Participants in the design sessions expressed agreement with the themes from the narrative review. Feedback from the participants led to the formation of 12 patient-facing video topics that could be split into four modules. The first module, ownership, focused on patient ownership of care and included topics like “What I can do at home to take medicines safely.” The second module, partnership, was intended to facilitate professional-patient partnership and communication. One of its topics was “What information doctors need from me during a visit.” We called the third module system. It educated patients on the roles of HCPs other than their primary care professional. One of its topics was “Who does what in the doctor’s office and why?” The fourth and final module was named learning and focused on the different ways that patients can find information about their medications, like “How to read and understand what is on my medicine bottles.” Table 3 shows the full list of video topics.

**Table 3:** Selected video topics.

Video topic list
Module 1: Ownership
Videos focused on patient ownership of their care
What I can do at home to take medicines safely
What I should do if I take medicines incorrectly
How to prepare for a visit with my doctor to make the most of the visit
Module 2: Partnership
Videos facilitating professional-patient partnership
How to talk to doctors about what I think about my medicines
How I can work together with my doctor to be safe and healthy
What information doctors need from me during a visit
Module 3: System
Videos about non-PCP professionals
Who does what in the doctor’s office and why
Outside of the doctor’s office, who else should I ask for help with my medicines?
What pharmacists can do to help me with my medicines
Module 4: Learning
Videos to help patients learn about managing their medications
How to find important information in handouts from my doctor’s office
How to read and understand what is on my medicine bottles
How to find good information from credible sources on my medicines

### 3.3 Survey of professionals and patients

A total of 44 professionals and 100 patients participated in the survey. The professionals included 27 physicians/faculty, 8 residents, 9 advanced practice providers, and 2 that did not specify. The non-residents averaged 9.6 years of experience practicing family medicine. The patients had a mean age of 51, were 67% female, and took 5.5 prescription medications on average.

**Figure 2:**
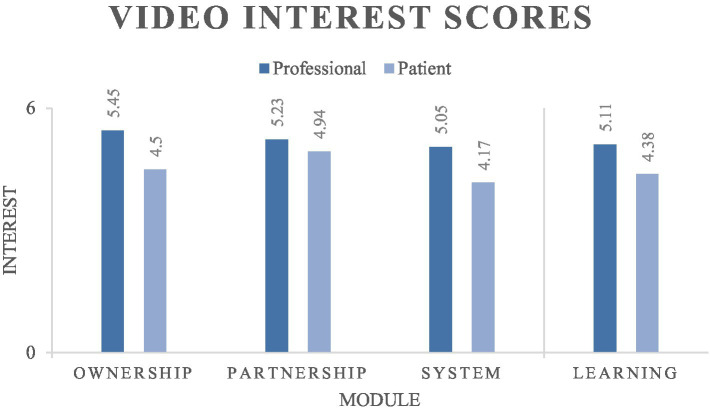
Interest scores by module.

The two-factor ANOVA that tested for differences between modules and roles showed that there was a significant difference in the interest expressed by role (i.e., professional or patient) (*p* < 0.001), with professionals reporting higher interest. The ANOVA did not find significant differences for module type (*p* = 0.094) or role-module interaction (*p* = 0.495). Figure 2 visualizes the mean interest scores for each module and Table 4 provides the results of the ANOVA test.

**Table 4:** Two-way ANOVA results.

Overall model	Sum of squares	df	Mean square	*F*	*p*
Overall model	82.74	7	11.82	4.63	<0.001^*^
Role	57.57	1	57.57	20.18	<0.001^*^
Module	18.34	3	6.11	2.14	0.094
Role × Module	6.83	3	2.28	0.80	0.495
Residuals	1563.25	548	2.85		

^*^Significant difference at *α* = 0.05.

Post-hoc testing via the Tukey method found significant differences between five role-module pairs. The most interesting is the difference between patient-partnership and patient-system (*p* = 0.038). Patient-system was the lowest scoring role-module pair. It also differed significantly from professional-ownership, professional-partnership, and professional-learning, but its comparison to patient-partnership is the only significant difference between two patient-module pairs. Thus, patients were significantly more interested in learning how to partner with their primary care professionals than they were in learning about other components of the healthcare system. The final significant comparison was between professional-ownership and patient-learning. Full post-hoc testing results are available as Supplementary material.

Qualitative analysis of the free-response answers revealed similarities and differences in the views of professionals and patients regarding medication safety education needs. Both groups emphasized the importance of medication adherence. Professionals stressed accountability, stating, “Patients need to be responsible and carry their medication list.” They also emphasized the increased importance for some types of medications, like antibiotics. Patients asked about correcting missed doses; for example, one patient asked, “Should I double my dose if I forget?” Both groups expressed concerns about medication safety. Professionals highlighted the need for education on “how to dispose of discontinued medications” while patients asked about “dangerous drug interactions” and “how to find out about recalled medications.” Health literacy also emerged as a shared concern. Professionals advocated for the use of visual aids, with one suggesting, “for my illiterate patients, it would be nice to have some kind of picture system to help manage medications.” In a similar vein, one patient asked, “What are my medicines actually prescribed for?” Lastly, professionals expressed a need for multilingual resources (“our patients speak Arabic, Dar, Pashto, Somali, Burmese, and more”), culturally competent material, and information that could help patients navigate the financial burden of medication (e.g., “GoodRx” and “social services”). Table 5 expands on the patient and provider perspective for each theme with sample quotes.

**Table 5:** Qualitative analysis of survey free-response.

Theme	Definition (patient & provider perspectives)	Sample patient quotes	Sample provider quotes
Medication adherence & responsibility	***For patients***: Understanding the importance of taking medications as prescribed, remembering doses, seeking guidance when unsure ***For providers***: Ensuring patients understand their prescriptions, emphasizing accountability, and reinforcing adherence strategies	“*To double or not when I forget*” “*No, I would ask a pharmacist*”	“*Patients need to be responsible and accountable and carry their medication list*” “*There are serious health consequences for not using their medications appropriately*”
Medication safety & proper use	***For patients***: Learning about potential drug interactions, safe disposal, and avoiding unnecessary medications ***For providers***: Educating patients on dangerous interactions, medication recalls, and proper discontinuation	“*How do you find out about recalled medication*?” “*Dangerous drug interactions*”	“*Adherence, benefits, over-the-counter meds (including antibiotics when they are not necessary)*” “*How to dispose of discontinued medication*”
Health literacy & medication label understanding	***For patients***: Struggling to read labels, understand dosages, and differentiate between medications ***For providers***: Addressing gaps in literacy by using simple language, visuals, and education tools to improve medication management	“*What my medicines are actually prescribed for*” “*What the medicine does and how it benefits and may have side effects*”	“*I would like to provide videos to help patients understand how to read labels on medicine bottles*” “*My patients do not read or write English. They do not understand how to read a medication label*”
Cultural & language barriers in healthcare	***For patients***: Facing challenges due to language differences, unfamiliarity with prescribed medicine, or cultural beliefs about health ***For providers***: Recognizing the need for culturally competent care, translation support, and accessible patient education	“*Alternative* ‘*medicine*’ *as holistic approach not relying on man’s concoctions adverse to God’s unadulterated designs that the body can use w/o all the-needless side effects, created of GREED/COMMERCIALISM*”	“*Our patients speak Arabic, Dari, Pashto, Somali, Burmese, Karen, Swahili, Kinyarwanda, and Nepali*” “*Hope you can make videos that target this population*”
Access to medication & financial assistance	***For patients***: Struggling with affordability, pharmacy access, and navigating insurance and discount programs ***For providers***: Helping patients access refills, find cost-saving options, and connect with social services		“*How to access refills, use of GoodRx or similar coupon sites*” “*Social services for additional assistance*”

## 4 Discussion

Recent studies have shown that well-designed education programs on patient safety can significantly improve patient outcomes (43). The present study used a co-design process to develop topics for short educational videos intended to improve medication safety by facilitating professional-patient partnership and shared decision-making. Each step of the process yielded important lessons. The narrative review found that knowledge-based hazards exist for both patients and healthcare professionals. Patients often lack the knowledge and skills to manage their medications, misunderstand their role in medication safety, and find it difficult to navigate the healthcare system. Professionals possess greater knowledge, but they also encounter knowledge-based barriers in the form of incomplete or inaccurate information about patients’ medication regimens and practices. The narrative review also identified opportunities for educational interventions: safety practices at home, communication and partnership, and how patients can learn about their medications. Our design sessions based on co-design methodologies validated those findings through the lived experiences of professionals and patients and helped us organize medication safety lessons into discrete video topics.

The professionals that participated in our video topic survey expressed significantly greater interest in the videos than patients did. In fact, the professionals’ interest score was higher than the patients’ for every module (illustrated in Figure 2). The patient work system offers a straightforward explanation for the difference: the videos represent additional work for patients. When deciding on whether they would be interested in the work, patient participants would weigh the utility of the videos with anticipated task and tool barriers in their work system (8). Conversely, professionals would only consider the utility of the videos, since the videos would not increase their workload. This explanation is supported by the post-hoc finding that professionals were significantly more interested in the ownership module than patients were in the learning module. A second, complementary explanation comes from information asymmetry: professionals may better understand the utility of the videos. Put another way—patients do not know what they do not know.

*Post-hoc* analysis also showed that patients are interested in learning to partner with their HCPs. Partnership was their highest scoring module and was significantly more interesting to patient participants than the system module. Patients understand that professionals possess great medical expertise. That sometimes leads them to offload their responsibilities for medication safety onto professionals (33, 34), but it also gives them a desire to harness that expertise through partnership. Information asymmetry provides a framework to understand that both actions (offloading responsibility and desiring partnership) are forms of patient reliance on professionals due to information imbalance (44). Several studies from our narrative review provide support that education can be used to change patients’ perception of their role from passive to engaged (19, 37, 38). That transformation is important because information sharing is difficult and can lead patients to feel demotivated (2). It requires effort and intention on the part of professionals and patients alike.

Education is one of many actions that HCPs perform to improve medication safety (40). Requesting information from patients and encouraging learning allows professionals to improve partnership and extend their influence beyond short visit times (19). It is important that they share that desire for patient ownership with their patients while understanding that education adds task barriers to the work performed in the PWS (8). Helping patients understand the importance of doing educational work pays dividends for HCPs because uninformed patients leave professionals to work with inaccurate or incomplete records and may offload their responsibilities for medication management to the HCPs. The resulting IA impedes shared decision-making and the co-creation of health outcomes (45). IA is reduced when professionals and patients share knowledge and goals openly (19).

### 4.1 Limitations

This study faces several limitations. Factors like sample size, sampling methods, and geographic concentration of participants limit generalizability. The professional sample for the survey is especially small at *N* = 44. All professional participants, all patient participants in the design sessions, and half of the patient participants in the survey were recruited from the clinics where we first implemented the videos resulting from this study (24). That aided our curriculum development and buy-in/implementation, but admittedly came at the expense of external validity. The large, public clinics where we recruited serve an extremely diverse patient population, but some of that diversity was lost by limiting the survey to English-speaking participants and only including Spanish speakers in the design sessions. Another limitation is that our measures do not include more concrete safety measures like adherence, intervention impact measures like behavioral change, or even an evaluation of partnership or communication practices. Our primary measure, interest in the videos, was an important part of the co-design process, but it does not necessarily lead to improved outcomes.

### 4.2 Future research

Future research in the field of medication safety and patient education should focus on several key areas to further enhance patient outcomes and healthcare practices. One promising direction should investigate the impact of interdisciplinary collaboration, particularly between pharmacists and primary care providers, on medication safety. Understanding how these collaborations can be optimized to enhance patient education and support can lead to more effective medication management strategies. The PWS provides a comprehensive framework for understanding the barriers patients face in managing their medications. Future research should continue to explore the PWS and its application in various healthcare settings to identify and mitigate these barriers. By addressing these patient-facing factors, healthcare professionals can develop targeted interventions that improve medication safety. Lastly, research linking partnership, communication, and shared decision-making to patient outcomes is critical.

## 5 Conclusion

Recent studies have shown that well-designed education programs on patient safety can significantly improve patient outcomes (43). This study used a co-design process to identify knowledge gaps that inhibit medication safety and develop educational topics that address them. Our narrative review revealed knowledge-based barriers to productive primary care encounters and education-based solutions. The design sessions with professionals and patients helped us organize the learning material into discrete topics. Lastly, our survey of professionals and patients showed that professionals are more interested in the videos than patients, but that patients are interested in learning to partner with their HCPs. Information asymmetry provides a framework to understand why some patients defer decision-making to professionals while also showing the greatest interest in the Partnership module. It also highlights why it is important for professionals to tell patients about their desire for patient ownership of care—engaged patients are less likely to abdicate their role in medication management and more likely to provide important information to professionals, who might otherwise work with incomplete records. To improve medication safety, patient education efforts should include a focus on how patients can partner with HCPs and be mindful of the work system barriers that patients will encounter while performing the educational work. Successful efforts stand to improve patient outcomes by reducing information asymmetry and enabling shared decision-making.

## Data Availability

The raw data supporting the conclusions of this article will be made available by the authors, without undue reservation.
