# Supplementary material for: What should patients learn? Co-designing patient education to improve medication safety, professional-patient communication, and partnership
Source: Front Med (Lausanne). 2025 Sep 10;12:1631606. doi: 10.3389/fmed.2025.1631606 (PMC12447905; doi:10.3389/fmed.2025.1631606)
Supplement: Supplementary file 1 [file Table_1.DOCX]

Post Hoc Comparisons – Role X Module

| Pair 1 | | | Pair 2 | | |  | | | | | |
| --- | --- | --- | --- | --- | --- | --- | --- | --- | --- | --- | --- |
| Role | Module | Role | | Module | Mean  Difference | | SE | df | t | p_tukey_ | Cohen’s d |
| Patient | Ownership | Patient | | Partnership | -0.347 | | 0.245 | 548 | -1.42 | 0.849 | -0.206 |
|  |  |  |  | System | 0.421 | | 0.245 | 548 | 1.72 | 0.675 | 0.249 |
|  |  |  |  | Learning | 0.211 | | 0.245 | 548 | 0.86 | 0.989 | 0.125 |
|  |  | Professional | | Ownership | -0.865 | | 0.308 | 548 | -2.81 | 0.095 | -0.512 |
|  |  |  |  | Partnership | -0.638 | | 0.308 | 548 | -2.07 | 4.435 | -0.378 |
|  |  |  |  | System | -0.456 | | 0.308 | 548 | -1.48 | 0.818 | -0.270 |
|  |  |  |  | Learning | -0.524 | | 0.308 | 548 | -1.70 | 0.686 | -0.310 |
|  | Partnership | Patient | | System | 0.768 | | 0.245 | 548 | 3.14 | 0.038* | 0.455 |
|  |  |  |  | Learning | 0.558 | | 0.245 | 548 | 2.28 | 0.308 | 0.330 |
|  |  | Professional | | Ownership | -0.518 | | 0.308 | 548 | -1.68 | 0.700 | 0.307 |
|  |  |  |  | Partnership | -0.290 | | 0.308 | 548 | -0.94 | 0.982 | -0.172 |
|  |  |  |  | System | -0.109 | | 0.308 | 548 | -0.35 | 1.000 | -0.064 |
|  |  |  |  | Learning | -0.177 | | 0.308 | 548 | -0.57 | 0.999 | -0.105 |
|  | System | Patient | | Learning | -0.211 | | 0.245 | 548 | -0.86 | 0.989 | -0.125 |
|  |  | Professional | | Ownership | -1.286 | | 0.308 | 548 | -4.18 | <.001* | 0.762 |
|  |  |  |  | Partnership | -1.059 | | 0.308 | 548 | -3.44 | 0.015* | 0.627 |
|  |  |  |  | System | -0.877 | | 0.308 | 548 | -2.85 | 0.086 | -0.519 |
|  |  |  |  | Learning | -0.945 | | 0.308 | 548 | -3.07 | 0.046* | -0.560 |
|  | Learning | Professional | | Ownership | -1.076 | | 0.308 | 548 | -3.49 | 0.012* | 0.637 |
|  |  |  |  | Partnership | -0.848 | | 0.308 | 548 | -2.75 | 0.109 | 0.502 |
|  |  |  |  | System | -0.666 | | 0.308 | 548 | -2.16 | 0.375 | 0.395 |
|  |  |  |  | Learning | -0.735 | | 0.308 | 548 | -2.39 | 0.251 | -0.435 |
| Professional | Ownership | Professional | | Partnership | 0.227 | | .360 | 548 | 0.63 | 0.998 | 0.135 |
|  |  |  |  | System | 0.409 | | .360 | 548 | 1.14 | 0.949 | 0.242 |
|  |  |  |  | Learning | 0.341 | | .360 | 548 | 0.95 | 0.981 | 0.202 |
|  | Partnership | Professional | | System | 0.182 | | .360 | 548 | 0.51 | 1.000 | 0.108 |
|  |  |  |  | Learning | 0.114 | | .360 | 548 | 0.32 | 1.000 | 0.067 |
|  | System | Professional | | Learning | -0.068 | | .360 | 548 | -0.19 | 1.000 | -0.040 |

* Significant difference at α = .05
